# Re: Park et al. “Association between job insecurity and cardiovascular disease among workers with type 2 diabetes mellitus”

**DOI:** 10.5271/sjweh.4293

**Published:** 2026-05-01

**Authors:** Johana Shiomara Paiva Suarez

**Affiliations:** 1 Escuela de Posgrado, Universidad Privada Antenor Orrego, Trujillo, Perú. [E-mail: johanapaiva1617@gmail.com]

We read with great interest the recent article by Park H et al (1), entitled “Association between job insecurity and cardiovascular disease among workers with type 2 diabetes mellitus”. The study provides important evidence on the impact of labor-related social determinants on cardiovascular health in a particularly vulnerable population: workers living with type 2 diabetes mellitus.

Previous research has consistently shown that psychosocial work-related factors, including job strain and employment insecurity, are associated with an increased risk of cardiovascular disease (2, 3). In this context, the study by Park and colleagues represents a valuable contribution to the field of occupational epidemiology. The use of a large national cohort derived from the Korean National Health Insurance Service, including more than 120 000 participants and a long follow-up period, strengthens the validity of the findings. Furthermore, the possibility of exploring differences according to sex and socioeconomic status aligns with the broader framework of social determinants of health (4).

Nevertheless, several aspects merit further consideration and could contribute to a more nuanced interpretation of the results.

First, the operational definition of job insecurity based on cumulative unemployment duration provides an objective and measurable indicator. However, this approach may not fully capture the broader spectrum of contemporary labor precariousness. Modern labor markets increasingly involve temporary contracts, underemployment, informal employment arrangements, and unstable contractual conditions. These forms of precarious employment may exert substantial effects on workers’ health but may not be adequately reflected by unemployment duration alone. Therefore, future studies may benefit from incorporating multidimensional measures of employment quality and labor instability.

Second, although the authors appropriately acknowledged the possibility of residual confounding in their discussion—particularly regarding unmeasured factors such as dietary habits, genetic predisposition, and limitations in capturing smoking behavior—additional sources of residual confounding may still be relevant. Factors such as depression, access to healthcare services, adherence to antidiabetic treatment, and dietary quality could simultaneously influence both employment stability and cardiovascular outcomes. This issue is especially important among individuals with diabetes mellitus, given their increased cardiometabolic vulnerability (5).

Third, the authors also recognized that the generalizability of their findings may be limited due to differences in occupational and cultural contexts across countries. In this regard, it is important to highlight that South Korea has a nearly universal health insurance system and a specific labor market structure. Consequently, extrapolating these findings to middle-income countries or settings characterized by high levels of labor informality should be done with caution. In such contexts, employment insecurity may manifest in more complex and heterogeneous ways that are not fully captured by administrative data (6).

Despite these considerations, the study has important implications for occupational health policies and clinical practice. The findings highlight the need for multidisciplinary strategies integrating labor policies, workplace health promotion, and interventions aimed at improving metabolic control among workers with chronic diseases. Addressing the interaction between adverse working conditions and noncommunicable diseases represents an emerging challenge for health systems and social protection policies worldwide (7).

Overall, this study significantly contributes to the growing body of literature examining the intersection between employment conditions and chronic disease outcomes. Future research conducted in more heterogeneous labor markets, particularly in settings with higher levels of informal employment, may help expand and strengthen the available evidence on the relationship between job insecurity and cardiovascular health.

We thank the authors for their valuable contribution and hope that these comments may stimulate further research in this important area of occupational and social epidemiology.
